# Regulation of Progenitor Cell Proliferation and Neuronal Differentiation in Enteric Nervous System Neurospheres

**DOI:** 10.1371/journal.pone.0054809

**Published:** 2013-01-23

**Authors:** Sokratis Theocharatos, David J. Wilkinson, Sarah Darling, Bettina Wilm, Simon E. Kenny, David Edgar

**Affiliations:** 1 Department of Cellular and Molecular Physiology, University of Liverpool, Liverpool, United Kingdom; 2 Institute of Child Health, University of Liverpool, Alder Hey Children's NHS Foundation Trust, Liverpool, United Kingdom; Temple University School of Medicine, United States of America

## Abstract

Enteric nervous system (ENS) progenitor cells isolated from mouse and human bowel can be cultured *in vitro* as neurospheres which are aggregates of the proliferating progenitor cells, together with neurons and glial cells derived from them. To investigate the factors regulating progenitor cell proliferation and differentiation, we first characterised cell proliferation in mouse ENS neurospheres by pulse chase experiments using thymidine analogs. We demonstrate rapid and continuous cell proliferation near the neurosphere periphery, after which postmitotic cells move away from the periphery to become distributed throughout the neurosphere. While many proliferating cells expressed glial markers, expression of the neuronal markers β-tubulin III (Tuj1) and nitric oxide synthase was detected in increasing numbers of post-mitotic cells after a delay of several days. Treatment of both mouse and human neurospheres with the γ-secretase inhibitor N-[N-(3,5-Difluorophenacetyl)-L-alanyl]-S-phenylglycine t-butyl ester (DAPT) reduced expression of the transcription factors Hes1 and Hes5, demonstrating inhibition of Notch signaling. DAPT treatment also inhibited progenitor cell proliferation and increased the numbers of differentiating neurons expressing Tuj1 and nitric oxide synthase. To confirm that the cellular effects of DAPT treatment were due to inhibition of Notch signaling, siRNA knockdown of RBPjκ, a key component of the canonical Notch signaling pathway, was demonstrated both to reduce proliferation and to increase neuronal differentiation in neurosphere cells. These observations indicate that Notch signaling promotes progenitor cell proliferation and inhibits neuronal differentiation in ENS neurospheres.

## Introduction

During vertebrate embryonic development, enteric nervous system (ENS) progenitor cells arising primarily from the vagal region of the neural crest migrate rostrocaudally along the gut, proliferating and differentiating to form the ganglia of the ENS [1], [2], [3]. Failure of this migration in humans results in Hirschsprung’s disease (HSCR), characterised by intestinal aganglionosis, which typically extends to a variable extent rostrally to include the internal anal sphincter, rectum and distal colon [4]. The absence of the ENS in the distal bowel causes a smooth muscle constriction that in turn gives rise to the megacolon seen in neonatal HSCR patients. Current treatment involves surgical resection of the aganglionic gut, but a high proportion of patients continue to experience postoperative morbidity [5], which may result from the small region of residual aganglionic distal bowel that always remains after surgery [4]. In recent years several groups have begun to assess the feasibility of using ENS progenitor cells for future use to provide a source of neurons to improve the function of this residual aganglionic gut [6].

We and others have isolated ENS progenitor cells from human and mouse gut and begun to characterise their properties both *in vitro* and after transplantation [7], [8], [9], [10]. Typically, the cells are grown in culture as aggregates known as neurospheres, by analogy with the neurosphere cultures previously described for stem cells derived from the central nervous system (CNS) [11], [12]. Both CNS and ENS neurospheres contain multipotent self-renewing neural progenitor cells and their neuronal and glial progeny [7], [11]. Significantly, ENS neurosphere transplantation into *ex vivo* explants of aganglionic embryonic gut restored a normal pattern of contractility [13].

It is essential to understand the mechanisms controlling progenitor cell proliferation, self-renewal and differentiation in neurospheres before the cells can be used safely for transplantation therapy, as continuing proliferation after transplantation could result in tumor formation. Clearly the niche provided by neurospheres in culture differs from that of ENS ganglia *in vivo*, and this difference is likely to be the reason for the proliferative behavior of the cells in neurospheres. It has been well documented that the proliferation and differentiation of a variety of neural progenitor cells can be regulated by the Notch signaling pathway [14], [15], [16]. While there is some evidence consistent with the need for Notch signaling during ENS development [17], [18], it remains to be established if Notch signaling can regulate the proliferation and differentiation of ENS progenitor cells.

As a prerequisite for future analysis of neurosphere cell behavior in vivo after transplantation, the work reported here characterizes cell proliferation and neuronal differentiation in ENS-derived neurospheres, and then investigates mechanisms controlling that behavior *in vitro*. We show that cells proliferate rapidly at or near the periphery of the neurosphere, after which postmitotic cells migrate throughout the neurosphere. While few cells expressing neuronal markers were found to be actively proliferating, expression of the markers increased several days after leaving the cell cycle. We furthermore demonstrate using chemical and siRNA inhibition that Notch signaling is necessary both for the maintenance of cell proliferation and suppression of neuronal differentiation in ENS neurospheres.

## Materials and Methods

### Ethics Statement

In accordance with the United Kingdom Animal (Scientific Procedures) Act of 1986, this study did not require a Home Office project license because no regulated procedures were carried out. Mice were humanely killed at a designated establishment by CO_2_ asphyxiation, which is an appropriate method under Schedule 1 of the Act. Ethical approval for the isolation of human ENS progenitor cells was given by the North West 3 Research Ethics Committee (Ref: 10/H1002/77). Written parental consent was obtained before samples were taken.

### Mouse ENS Neurosphere Preparation

Time mated CD-1 mice (Charles River Laboratories, UK) were sacrificed 11.5 days post-coitum by inhalation of increasing concentrations of carbon dioxide. The preparation of ENS neurospheres has been described in detail previously [7], [13]. Briefly, dissected ceca were incubated with 0.05% (w/v) trypsin (Sigma Aldrich, UK) in Dulbecco's phosphate buffered saline (PBS, Invitrogen, Life Technologies, UK) for 15 min at 37°C. After mechanical dissociation, 2–3×10^6^ cells were transferred to 60 mm non-adherent culture dishes (Sterilin, ThermoFisher Scientific, UK) in 4 ml Dulbecco’s modified Eagle medium (1 mg/ml glucose), 100 U/ml penicillin and 100 µg/ml streptomycin (Invitrogen, Life Technologies, UK), 2 mM L-glutamine (Invitrogen, Life Technologies, UK), 2% v/v chick embryo extract (Sera Laboratories Int., UK), 1% (v/v) N1-supplement (Sigma-Aldrich, UK), 0.05 mM 2-mercaptoethanol (Sigma-Aldrich, UK), 20 ng/ml, EGF (Sigma-Aldrich, UK) and 20 ng/ml FGF2 (Autogen Bioclear, UK). The culture medium was replaced every 96 h, and after 2 weeks the suspended neurospheres had reached diameters of about 100 µm.

### Human ENS Neurosphere Preparation

Human neurospheres were generated from the ganglionic colon of neonates undergoing elective abdominal surgery as previously described [7], [13]. Briefly, after removing the mucosa and submucosa from 1 cm^2^ full thickness gut samples, the muscle layers were mechanically disrupted into 1–2 mm^2^ pieces. This was followed by 1 h incubation with 0.5% (w/v) collagenase and 0.5% (w/v) dispase (Gibco, Life Technologies, UK) in PBS at 37°C before trituration. The incubation step was repeated 2–4 times with fresh enzyme solutions until a single cell suspension was obtained. The cell suspension was then cultured under the same conditions as used to generate mouse neurospheres. The human neurospheres were used when they had reached either the secondary or tertiary generation, both of which have been previously characterized [19].

### Formation of Chimeric Neurospheres

A single-cell suspension was prepared by dissociation of 2–3 week old mouse neurospheres by trypsinization (0.05% w/v trypsin in PBS for 10 min) and trituration. Constitutive expression of enhanced green fluorescent protein (eGFP) under control of the spleen focus-forming viral (SFFV) promoter was by Lentiviral transduction. After the cells had begun to express eGFP (2- days), chimeric neurospheres were produced by centrifuging 5×10^3^ labeled cells at 150 g onto aliquots of unlabeled neurospheres taken from the same batch as that used to obtain cells for viral transduction. The chimeric neurospheres were maintained in suspension culture for a further 96 h before fixation.

### Immunostaining

Neurospheres were transferred into Shandon Cryomatrix (Thermo Fischer Scientific, UK) and stored at −80°C until 8 µm serial frozen sections were prepared by cryostat. For single cell analysis, the neurospheres were dissociated by trypsinization and trituration. 5×10^3^ aliquots of cells were allowed to attach to Permanox 8-chamber culture slides (Sigma Aldrich, UK) for 3 h before fixation.

Neurosphere sections and single cells were fixed with 4% (w/v) paraformaldehyde (PFA) followed by permeabilization with 0.25% (w/v) Triton X-100 in PBS (Sigma-Aldrich, UK). After rinsing and blocking with 2% (w/v) bovine serum albumin (Sigma Aldrich, UK) in PBS, primary antibodies were applied at the following dilutions in the blocking buffer: rabbit anti-p75 (Abcam, UK) 1∶500; rabbit anti-GFAP (DAKO, UK) 1∶1000; mouse anti-GFAP (Sigma-Aldrich, UK) 1∶1000; goat anti-Sox10 (Santa Cruz, USA) 1∶100; mouse anti-Tuj1 (Abcam, UK) 1∶500; rabbit anti-S100 (Abcam, UK) 1∶800; rabbit anti-NOS (Abcam, UK) 1∶800. Isotype controls were performed with the same concentrations of non-immune antibodies. After incubation overnight at 4°C, samples were rinsed, followed by 2 h incubation with the appropriate secondary antibodies diluted 1∶1000 in blocking buffer (all from Invitrogen, Life Technologies, UK). All primary antibodies react with mouse and human antigens.

Assessment of immunoreactivity was made using standard fluorescence or confocal microscopy where specifically stated. Counting of immunopositive and EdU-positive cells was undertaken in >5 random optical fields across each chamber using a standard fluorescence microscope and 40x oil objective.

### BrdU and EdU Incorporation

Two to 3 week old mouse neurospheres were incubated in culture medium containing 10 µM bromodeoxyuridine (BrdU) for the times shown. For BrdU staining, frozen fixed neurosphere sections were treated with 4 M HCl for 15 min and rinsed with distilled water prior to permeabilization and immunostaining for nuclear BrdU (DAKO, UK). For cells dissociated from neurospheres, proliferation was assessed by incubation with 10 µM ethynyldeoxyuridine (EdU) for 1 h immediately before dissociation to single cells (this procedure does not use HCl treatment and so helps preserve the morphology of cells). Dissociated cells were allowed to attach briefly to Permanox culture slides before processing according to the manufacturer’s instructions to visualize nuclear EdU by the binding of the azide group of the Click-it^®^ Alexa594 fluorophore to the alkyne group of EdU (Click-it EdU Imaging Kit, Invitrogen, Life Technologies, UK).

### Inhibition of Notch Signaling

Preliminary experiments investigating the effects of Notch inhibition used the γ-secretase inhibitor N-[N-(3,5-Difluorophenacetyl)-L-alanyl]-S-phenylglycine t-butyl ester (DAPT, Sigma Aldrich, UK), dissolved in dimethyl sulfoxide (DMSO). An equal volume (5 µl) of DMSO was applied to control dishes. For determination of proliferation and expression of neuronal and glial markers, cells were dissociated from the DAPT-treated neurospheres and controls by trypsinization and trituration, after which they were allowed to attach to Permanox chamber slides in culture medium before fixation with 4% (w/v) PFA.

siRNA knockdown of RBPjκ was performed with human neurosphere cells attached to Permanox chamber slides using the following oligomers (Qiagen, UK): HsRBPJ_1 (TAGGGAAGCTATGCFAAATTA); HsRBPJ-2 (GTGGCTGGAATACAAGTTGAA); HsRBPJ_3 (CACGGTATTATAGTACACCTT). The control oligomers used were the Qiagen All Stars Human Cell Death Control^®^ and All Stars Negative Control^®^. Transfection was performed according to the manufacturer’s instructions using a HiPerFect^®^ transfection kit (Qiagen, UK). The transfection reagent was used at a concentration of 3 µl/ml with a final oligomer concentration of 10 nM. Determination of Tuj1 expression and EdU incorporation was performed after 96 h as described above.

### qPCR

RNA was extracted using the Trizol^®^ Reagent (Invitrogen, Life Technologies, UK) according to the manufacturer’s instructions, using 20 µg/µl glycogen (Invitrogen, Life Technologies, UK) as carrier. Extracted RNA was treated with 1 U/µl RQ1 DNase (Promega, UK) before cDNA synthesis with Superscript^®^ III reverse transcriptase (Promega, UK). The qPCR reaction using a Corbett Rotor-Gene RG-3000 thermal cycler (Qiagen, UK), was with KAPA-SYBR^®^ hot start master mix (KAPA BIOSYSTEMS, UK).

The primers used to determine levels of mouse Hes1 and Hes5 mRNA were: Hes1: GCACAGAAAGTCATCAAAGCC forwards, TTGATCTGGGTCATGCAGTTG reverse; Hes5: AGTCCCAAGGAGAAAAACCGA forwards, GCTGTGTTTCAGGTAGCTGAC reverse, β-actin: CGTTGACATCCGTAAAGACC forwards, CAGGAGGAGCAATGATCTTGA reverse. For human RBPjκ mRNA, a QuantiTect^®^ kit primer assay for RBPjκ (QT01680049, Qiagen, UK) was used according to the manufacturer’s protocol. PCR products were analysed by agarose gel electrophoresis, melting curves and sequencing. Expression of the target genes relative to β-actin mRNA was determined using the comparative C_t_ method [20].

### Data Analysis

Statistical analysis was performed using GraphPad Prism Version 5 (GraphPad Software, USA). Significance was determined by Student t-test or ANOVA as indicated in figure legends. Results are expressed as mean ± SEM.

## Results

### Analysis of Neurosphere Cell Proliferation and Migration

**Figure 1:**
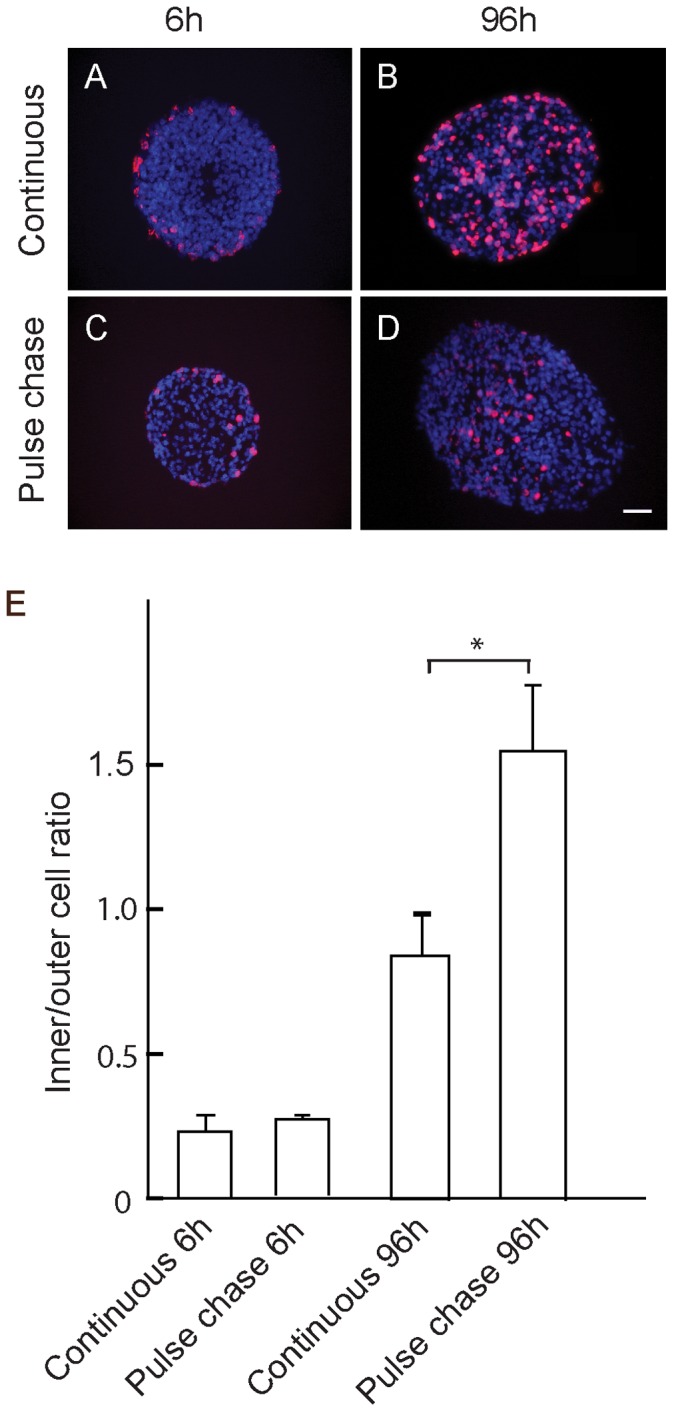
Cell proliferation in mouse neurospheres after continuous labeling and pulse-chase with BrdU. A, B: Continuous labelling with 10 µM BrdU after 6 h (A) and 96 h (B) culture. C, D: Labelling after 1 h pulse of 10 µM BrdU, neurospheres immediately after the pulse (C), and 96 h after chase in absence of BrdU (D). Images show immunostaining for BrdU of sections taken through the equatorial region of the neurospheres, counterstained with DAPI. E: Ratio of BrdU stained nuclei in the inner 50%/outer 50% of the area of the sections. Values are means±SEM (n>5) from 2 independent experiments. *p<0.01. Scale bar = 25 µm.

Two to 3 week old primary mouse neurospheres were cultured for up to 96 h in medium containing 10 µM BrdU. Photomicrographs were recorded of sections cut through the centre of each neurosphere, which were identified as the serial sections with the greatest diameter. After 6 h incubation, BrdU incorporation was restricted to a few cells at or near the periphery of the neurosphere (Fig. 1A). In contrast, by 96 h many of the nuclei had incorporated BrdU (Fig. 1B). Moreover, the labeled cells were distributed uniformally throughout the neurosphere (Fig. 1E).

Pulse-chase analysis confirmed initial incorporation of BrdU into cells at or near the neurosphere periphery immediately after a 1 h pulse (Fig. 1C). However, after a 96 h chase, very few weakly labeled cells could be detected at the periphery while strongly labeled nuclei were now distributed toward the centre of the neurosphere (Fig. 1D, E). Images produced during the chase in the absence of BrdU showed that the level of labelling of nuclei at the periphery of the neurosphere gradually decreased with time to become virtually undetectable by 96 h, while strongly labeled nuclei were located nearer the centre of the neurosphere (Fig. 1D and Fig. S1). These observations indicate that cells remaining at or near the neurosphere periphery continue to proliferate while postmitotic cells move from the periphery to become distributed throughout the neurosphere.

**Figure 2:**
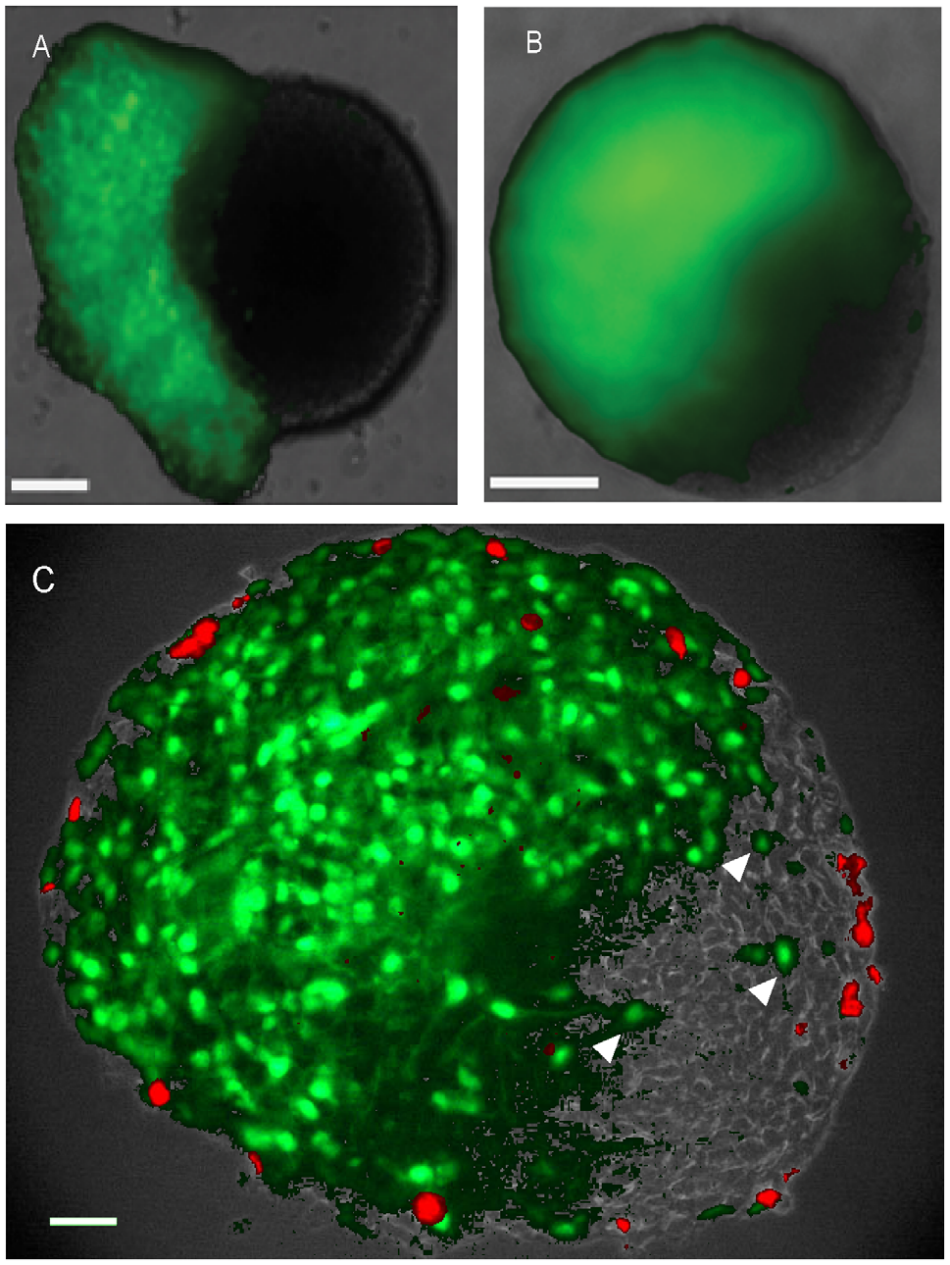
Cell migration and proliferation in chimeric mouse neurospheres. eGFP expressing neurosphere cells were centrifuged onto unlabeled intact neurospheres to produce chimeric neurospheres. Wholemount fluorescence images of living neurospheres taken (A) after 24 h and (B) after 96 h culture, showing green eGFP fluorescence: note that after 96 h the boundary between labeled and unlabeled cells has become diffuse. C, 8 µm equatorial section of typical chimeric neurosphere after 96 h culture, at the end of which the neurospheres had been incubated with 10 µM BrdU for 1 hour before fixation. Note that eGFP cells have migrated into the unlabeled half of the chimeric neurosphere (C, arrow heads), and that BrdU labelling (red) is restricted to the periphery of the chimeric neurosphere. Scale bars = 100 µm.

To demonstrate directly that cells move within the neurospheres, we constructed chimeric neurospheres in which about half the cells were labeled with the constitutively expressed eGFP (Fig. 2A). After 96 h, the initially sharp boundary between GFP -positive and –negative cells became indistinct in living whole mount preparations (Fig. 2B). Analysis of fixed frozen sections at this time point showed that eGFP -positive cells had migrated into the unlabeled neurosphere halves (Fig. 2C). Furthermore, cell proliferation was again restricted to the periphery of both halves of the chimeric neurosphere while being absent from the interface between eGFP –positive and –negative cells (Fig. 2C). These observations demonstrate directly that there is indeed cell movement within the neurosphere.

### Relationship between Cell Proliferation and Differentiation

**Figure 3:**
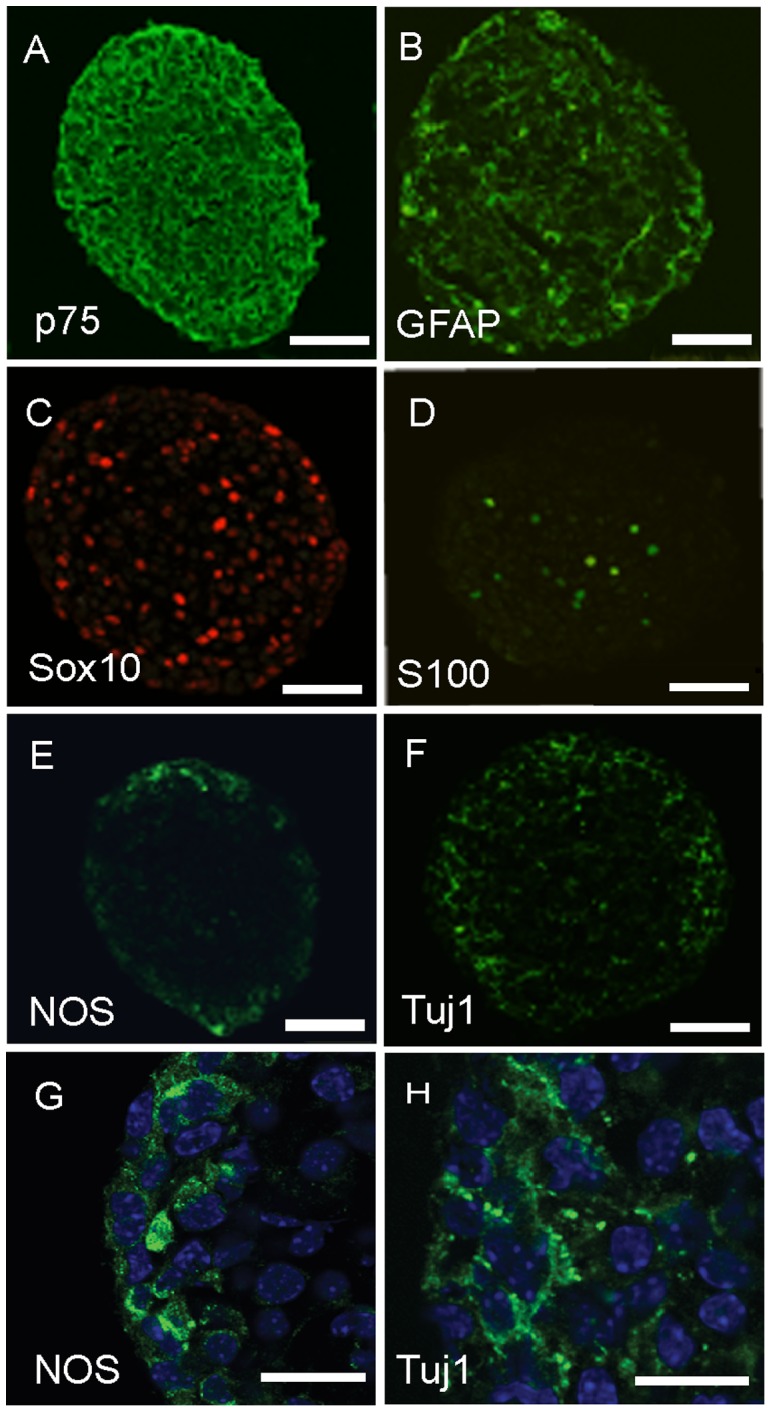
Expression of progenitor, glial and neuronal markers by mouse neurosphere cells. Primary neurospheres were fixed after 15 days culture and 8 µm equatorial sections produced. Confocal immunofluorescence images are shown. G and H are higher magnifications of the peripheral immunofluorescence for NOS and Tuj1 shown in E and F, respectively. Note that NOS and TuJ1 immunofluorescence is located both in cell bodies and fibers (G,H, arrow heads). Scale bars: A–F: 25 µm; G,H: 10 µm.

Confocal immunofluorescence of equatorial neurosphere sections showed that the majority the cells expressed p75, consistent with their neural crest origin (Fig. 3A), whereas cells expressing the ENS progenitor and glial marker Sox10 were distributed sparsely throughout the neurosphere (Fig. 3C), as were the glial markers GFAP and S100 (Fig. 3B and D). In contrast, immunoreactivities of the neuronal markers, neuronal Class III β-Tubulin (Tuj1) and nitric oxide synthase (NOS) were mainly located near the neurosphere periphery and only a few sporadic cells or fibers were seen throughout the neurosphere (Fig. 3E and F). Higher power confocal microscopy showed that the subcellular localization of NOS and Tuj1 immunoreactivity was difficult to define, as these neuronal markers appeared to be in both cell bodies and fibers (Fig. 3G and H).

**Figure 4:**
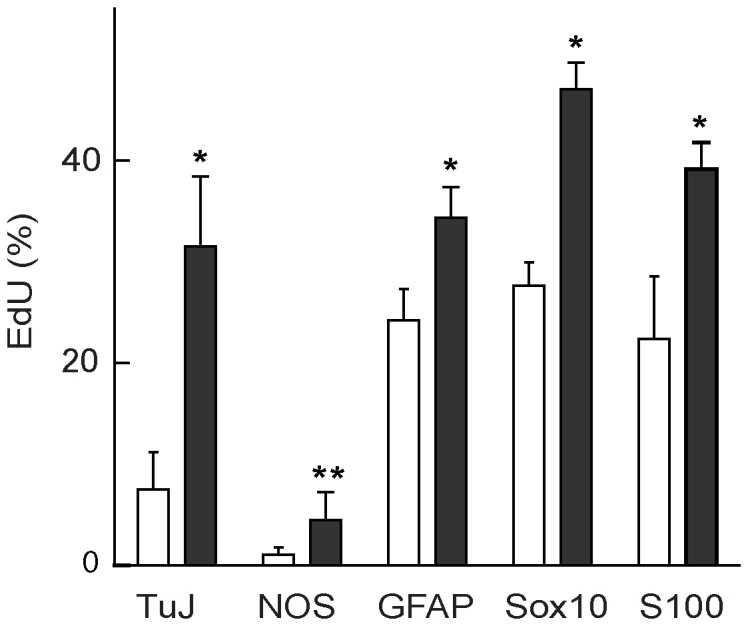
Analysis of proliferation of mouse neurosphere cells expressing neuronal and glial markers. Neurosphere cells were dissociated and allowed to attach to chamber slides immediately after a 1 h pulse of 10 µM EdU (open columns), or after a 96 h chase in the absence of EdU (closed columns). The vertical axis shows the percentage of cells positive for specific phenotypes that had also incorporated EdU. Error bars represent SEM (n = 3 separate experiments). A two-tailed t-test was performed for differences between before and after chase (open and closed columns) for each marker. * p<0.05; ** p<0.075.

Because it was difficult to quantitate numbers of Tuj1- and NOS- expressing cells in the neurospheres (see Fig. 3), we dissociated the neurospheres to single cells which were then allowed to attach to adhesive substrates in order to double label and count cells for marker expression and proliferation demonstrated by incorporation of the thymidine analog EdU (Fig. S2). Very few cells expressing the neuronal markers Tuj1 (<8%) or NOS (<2%) had incorporated EdU immediately after the pulse (Fig. 4). Significantly, 96 h after EdU labeling, the proportion of labeled cells expressing Tuj1 and NOS neuronal markers had increased about 5-fold (Fig. 4), indicating that postmitotic progenitor cells differentiate to acquire a neuronal phenotype with a delay of several days. Although increased numbers of labeled cells expressing the glial markers 96 h after EdU labeling were also found, this increase was less than that found for the neuronal cells, due in part to the relatively high proportion of glial cells that were expressing these markers during or immediately after the labeling (Fig. 4).

### Role of Notch Signaling in Neurosphere Cell Proliferation and Differentiation

**Figure 5:**
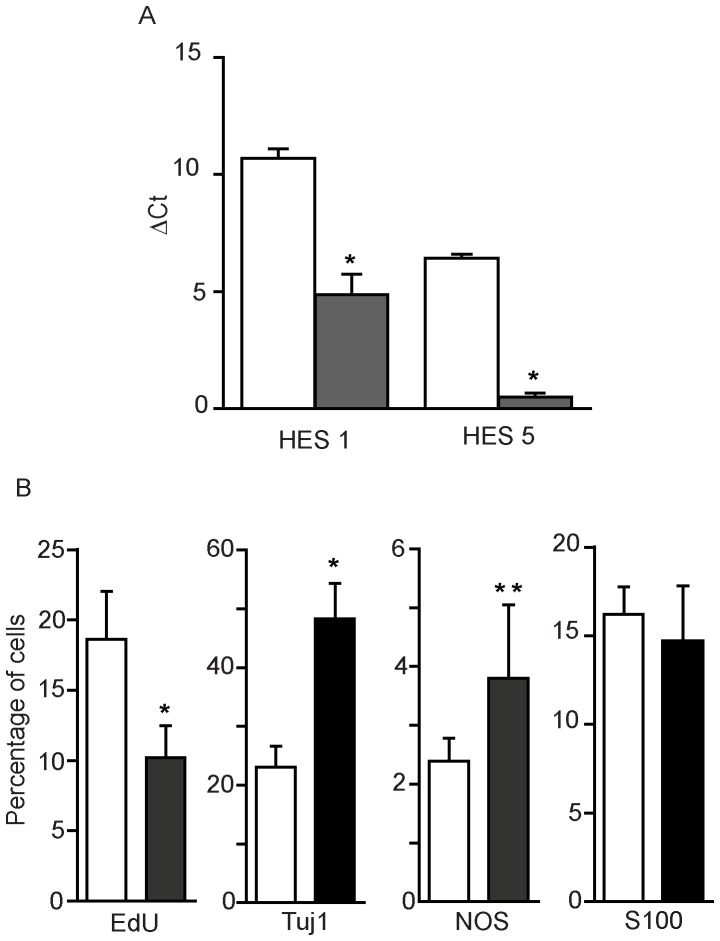
Effects of DAPT-mediated γ-secretase inhibition on cell proliferation and expression of neuronal and glial markers by mouse neurosphere cells. After 2 weeks culture mouse neurospheres were cultured for a further 96 h in the presence of 20 µM DAPT (shaded columns) or DMSO solvent control (clear columns). A: Levels of Hes1 and Hes5 mRNA determined by q-PCR. Columns show the ΔC_t_ values, normalised to β-actin levels (± SEM, means of 3 individual experiments). B: Expression of neuronal and glial markers. Cells dissociated from the neurospheres were allowed to adhere to Permanox slides before fixation and staining for EdU, Tuj1, NOS and S100. Nuclei were counter-stained with DAPI. Fluorescent cells were counted in 5 random optical fields in each chamber using a 40 x oil objective. Error bars are ± SEM, (values from 3–5 experiments). A two-tailed t-test was performed for differences between open and closed columns for each marker. *p<0.01.

**Figure 6:**
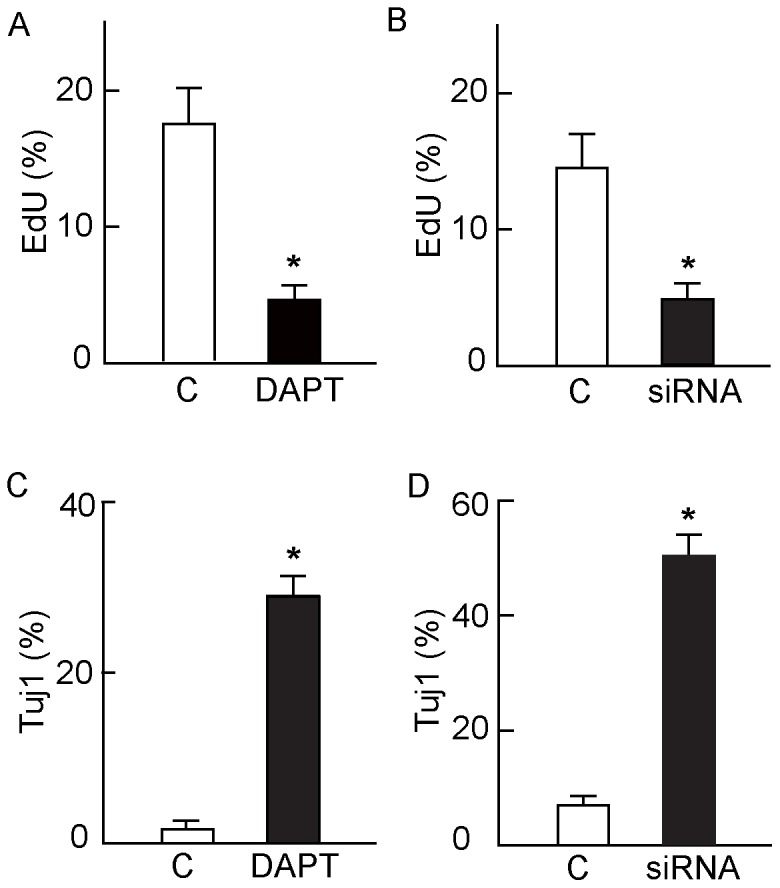
Effects of DAPT and RBPjκ siRNA-mediated Notch inhibition on human neurosphere cell proliferation and neuronal differentiation. A and C: Cells dissociated from human neurospheres were allowed to adhere to Permanox tissue culture slides and cultured with and without 20 µM DAPT. After 96 h the cells were fixed and stained for Tuj1, and nuclei counterstained with DAPI. Fluorescent cells were counted in 5 random optical fields in each chamber. Error bars are ± SEM, (values from 3–5 experiments). A: Significantly fewer DAPT-treated cells incorporated EdU than controls (* p<0.001 ANOVA) while expression of Tuj1 (C) was markedly increased over control levels (* p<0.001 ANOVA). B and D: The effect of siRNA knockdown on the human neurosphere cells prepared and cultured under the same conditions as in A and C. Relative to controls transfected with an irrelevant siRNA, after 96 h siRNA transfection with HsRBPJ_3 siRNA specific for RBPjκ had (B) a lower rate of proliferation (* p<0.001, ANOVA) and (D) increased expression of Tuj1 (* p<0.001, ANOVA). Error bars are ± SEM (n = 3).

Initial experiments implicating Notch signaling in the proliferation and differentiation of neurosphere cells utilized the chemical inhibitor of γ-secretase DAPT, which blocks Notch signaling by inhibiting the cleavage of the Notch intracellular signaling peptide NICD [21]. Incubation of mouse neurospheres for 96 h with DAPT reduced mRNA levels of the transcription factors Hes1 and Hes5 which are downstream targets of NICD in the canonical Notch signaling pathway (Fig. 5A and Fig. S3). The level of Hes1 was reduced by 50%, and Hes5 decreased by almost 90% relative to controls, indicating an effective block of Notch signaling. Significantly, γ-secretase inhibition reduced neurosphere cell proliferation to 55% of controls (Fig. 5B), while the numbers of Tuj1-positive and NOS-positive neuronal cells increased. In contrast, the numbers of cells expressing the glial cell marker S100 did not change significantly (Fig. 5B). Similarly, DAPT treatment reduced the proliferation of cells dissociated from human neurospheres while increasing the numbers of Tuj1-positive cells present (Fig. 6A and 6C).

Although these results are consistent with a role for Notch signaling in the regulation of ENS progenitor cell proliferation and differentiation in neurospheres, they do not prove it because inhibition of γ-secretase may affect other signaling pathways with which it is involved [21]. In order to demonstrate that Notch signaling does indeed regulate neurosphere cell proliferation and differentiation, we investigated the effects of siRNA knock-down of RBPjκ (a key component of Notch signaling [22]). The effectiveness of the knockdown was determined by a reduction of mRNA levels of RBPjκ after siRNA treatment using the HsRBPJ_3 oligomer to about one third of control levels in human neurosphere cells (Fig S4). Similar to the effects of DAPT treatment of these cells, siRNA knockdown of RBPjκ with the HsRBPJ_3 siRNA resulted in a three-fold reduction of proliferation (Fig. 6B) and greater than five-fold increase in expression of the early neuronal marker Tuj1 (Fig. 6D). Similar decreases were found with the other two siRNAs directed against RBPjκ, HsRBPJ_1 and HsRBPJ_2 (data not shown). Taken together, these results show that Notch signaling is necessary for progenitor cell proliferation and inhibition of neuronal differentiation in ENS neurospheres.

## Discussion

Although neurosphere culture offers a potential method for the amplification of ENS progenitor cells for future transplantation therapies in HSCR [6], [8], [13], there have been no studies into why the cells proliferate rapidly in neurospheres, nor why some of them cease proliferation and differentiate into postmitotic neural cells. This knowledge will be necessary for future work to ensure that ENS cells do not proliferate uncontrollably after transplantation, and also to optimise therapy by promoting the differentiation of the most functionally effective neuronal subtypes. Our investigations here demonstrate that rapid cell proliferation occurs at or near the periphery of ENS neurospheres, after which postmitotic cells move throughout the neurosphere. Neuronal differentiation occurs after the progenitor cells cease proliferation, and both proliferation and neuronal differentiation of ENS neurosphere cells are regulated by Notch signaling.

ENS progenitor cells have previously been isolated and cultured by a variety of methods that differ in the sources of cells, methods of isolation and tissue culture techniques used to grow them [7], [8], [9], [10]. These variations may well be responsible for differences in the properties of the cells reported. For example, it is known that ENS progenitor cells isolated from developing gut have more restricted differentiation potential as they mature [23]. Furthermore it has been shown recently that neurosphere-like bodies isolated from submucosal and myenteric regions of the bowel contain distinct subpopulations of cells that differ in their functions and phenotypes [9]. Thus, neurosphere-like bodies derived from postnatal human submucosal tissue have been demonstrated to comprise a majority of mesenchymal non-neural cells and their progenitors, necessitating cell purification techniques to enrich the small subpopulation of ENS cells present [9], [24]. In contrast, neurospheres derived from embryonic mouse gut and from the myenteric region of postnatal human bowel give rise to neurospheres in which the majority of cells express the p75 marker (see Fig. 4A), consistent with their neural crest origin [7], [13], [19].

We previously demonstrated the presence of multipotent progenitor cells in neurospheres derived both from embryonic mouse gut and from postnatal human bowel [7], [19]. While this work showed that the progenitor cells retain similar differentiative and proliferative properties over a period of months in culture [7], [19], ENS neurosphere cell proliferation and its relationship to differentiation has not been investigated in any detail. We demonstrate here by pulse-chase experiments with thymidine analogs that a brief exposure to BrdU results in a rapid initial labelling of cells at or near the neurosphere periphery. Although the peripheral location of BrdU incorporation may be due to its inability to penetrate completely into the neurosphere during this brief one hour labelling period, it is significant that the level of labelling of the peripheral cells gradually declines with time after the pulse of BrdU, while heavily labeled cells are found distributed throughout the neurosphere (see Fig. 1). Given our demonstration that there is considerable mixing of cells in chimeric neurospheres (see Fig. 2), then the simplest interpretation of these observations is that the progeny of cells that initially divided at the neurosphere periphery slowed or stopped proliferation as they migrated throughout the neurosphere, whereas progeny remaining at the periphery continued to proliferate.

The spatial pattern of cell proliferation and its relationship to cell migration in ENS neurospheres is reminiscent of that in the CNS in which progenitor proliferation in germinal zones located peripherally is followed by the migration of postmitotic cell progeny to deeper layers of the CNS [25]. Furthermore, evidence has been presented from *in vivo* studies that extraganglionic post-mitotic ENS progenitor cells migrate into ganglia in the adult nervous system [26]. Thus, progenitor cell behavior in ENS neurospheres mimics that of both CNS and ENS progenitors *in vivo*. Future analysis of the relationship between proliferation and migration in neurospheres will help to establish the mechanisms coordinating this general behavior of neural progenitor cells.

Our earlier studies provided preliminary evidence that the proportion and phenotypes of neurons in secondary and tertiary ENS neurospheres remain constant, implying that progenitor cell proliferation and differentiation of their progeny are closely linked [19]. We show here that very few cells that had incorporated the thymidine analog also expressed the neuronal markers Tuj1 or NOS immediately after the pulse. However, after a chase period of 96 h the numbers of dual-labeled cells had increased significantly, indicating that neuronal differentiation occurs after the neurosphere cells had withdrawn from the cell cycle. This conclusion is consistent with a detailed earlier study which clearly demonstrated a close relationship between the timing of withdrawal of ENS progenitor cells from the cell cycle and the differentiation of specific neuronal phenotypes *in vivo* [27]. Thus, similar control mechanisms may be responsible for the proliferative and differentiative behavior of ENS neural progenitor cells both *in vivo* and in neurospheres *in vitro*.

Injury to the postnatal ENS results in a mitotic response of cells which may either be glial in origin [28], [29], or possibly be a small number of quiescent progenitor cells remaining in or close to the ENS ganglia [26]. These proliferating cells have the properties of multipotent neuronal and glial progenitor cells, although transplantation studies have shown that the environment *in vivo* can affect their differentiation by biasing it towards a glial phenotype [28]. It is of interest to note that while neuronal differentiation in neurospheres occurred following a delay after cell proliferation, a larger proportion of neurosphere cells expressing glial markers were labeled with the thymidine analog immediately after the short pulse. This reflects the previous observations indicating that neural crest-derived cells expressing glial markers are able to proliferate *in vitro* [28], [29]. Indeed, it is now well established that GFAP-expressing cells are multipotent progenitors for both neurons and glia in the developing CNS [30]. In this context it should be noted that the procedure used to isolate ENS cells during the production of neurospheres constitutes an injury which is likely to contribute to stimulation of cell proliferation in ENS neurospheres [28], [29].

Notch receptors and their ligands are expressed in the ENS [17], [31], and evidence for a role for Notch signaling during ENS development has been provided by showing that inhibition of the Notch pathway resulted in defective ENS development in embryonic mice, associated with premature neurogenesis and reduction in ENS progenitor cells [17]. We show here that blocking the canonical Notch signaling pathway in ENS neurosphere cells by either siRNA directed against RBPjκ or chemical inhibition with DAPT inhibits progenitor cell proliferation while increasing the numbers of cells expressing the neuronal marker Tuj1. Although both embryonic mouse and neonatal human ENS neurosphere cells display Notch-dependent proliferation and inhibition of neuronal differentiation, the Notch signaling in neurospheres did not result in a permanent shift in ENS progenitor cell potential from neurogenic to gliogenic, as has been reported for other neural crest cell derivatives [32].

It remains to be established which other factors modulate the Notch-dependent maintenance of progenitor cell self-renewal *in vivo* and *in vitro*. In this context it is important to note that sonic hedgehog has recently been shown to increase Notch signaling in ENS progenitor cells via induction of the Notch ligand DLL3 [18]. Furthermore, evidence has been presented indicating that Notch signaling may be compromised in HSCR bowel [33]. Our in vitro work presented here provides the basis for future experiments to determine if Notch signaling regulates ENS progenitor cell behavior after transplantation, and if this can be manipulated to improve the outcome for HSCR patients.

## Supporting Information

Figure S1 **Neurosphere cell labeling during 4 day chase after a 1** **h pulse of BrdU.** Primary mouse neurospheres previously cultured in suspension for 15 days were labeled with a 1 h pulse of 10 µM BrdU. After BrdU removal and washing, an aliquot of the neurospheres was fixed and the remaining neurospheres were then cultured further, removing aliquots for fixation at 1, 2 and 4 days. BrdU immunostaining (red) was performed on 8 µm cryostat sections taken from the equatorial region of the neurospheres, followed by counterstaining of nuclei by DAPI (blue). Scale bar = 25 µm.
(TIF)

Figure S2 **Double labeling of neurosphere cells for neural cell markers and EdU incorporation.** Primary mouse neurospheres previously cultured for 15 days under non-adherent conditions were labeled with a 1 h pulse of 10 µM EdU. The neurospheres were then dissociated and allowed to attach after which they were fixed and permeabilized before immunostaining for the neural cell markers shown and processing to reveal EdU incorporation. The montages shown were constructed in Adobe Photoshop from 3 separate images captured to demonstrate the EdU incorporation, immunofluorescence and phase contrast images. Scale bars = 25 µm.
(TIF)

Figure S3 **Agarose gel electrophoresis of qPCR products after DAPT treatment of neurospheres.** The PCR products obtained from the experiment in Fig. 5A was electrophoresed in 2% agarose gels. Calibration standards (bp) are shown on the left hand side of each gel. PCR product sizes were: Hes1 = 354 bp, Hes5 = 183 bp and 269 bp and β-actin = 143 bp. The DNA in each excised band was sequenced to confirm PCR product identity; the double bands for Hes5 represent two splice variants amplified by the primer pair used.
(TIF)

Figure S4 **Confirmation of RBPjκ knockdown in human neurospheres.** Mature 2^nd^ to 3^rd^ passage human neurospheres were dissociated and cultured on fibronectin coated chamber slides for 96 h. The dissociated cells were transfected with HsRBPJ_3 siRNA knockdown specific for RBPjκ or a corresponding negative control. Levels of RBPjκ were determined by qPCR. Columns show the normalised ΔC_t_ values (± SEM, n = 3). * = P<0.01.
(TIF)
