# Supplementary figures and images for: Regulation of Progenitor Cell Proliferation and Neuronal Differentiation in Enteric Nervous System Neurospheres
Source: PLoS One. 2013 Jan 23;8(1):e54809. doi: 10.1371/journal.pone.0054809 (PMC3553067; doi:10.1371/journal.pone.0054809)

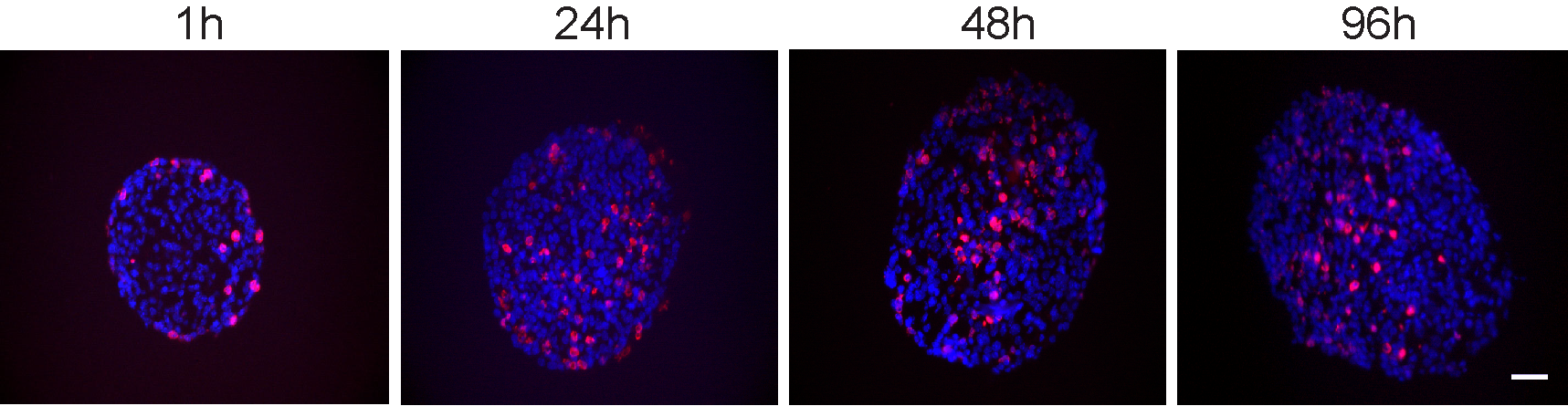

Supplement: Figure S1 — Neurosphere cell labeling during 4 day chase after a 1 h pulse of BrdU. Primary mouse neurospheres previously cultured in suspension for 15 days were labeled with a 1 h pulse of 10 µM BrdU. After BrdU removal and washing, an aliquot of the neurospheres was fixed and the remaining neurospheres were then cultured further, removing aliquots for fixation at 1, 2 and 4 days. BrdU immunostaining (red) was performed on 8 µm cryostat sections taken from the equatorial region of the neurospheres, followed by counterstaining of nuclei by DAPI (blue). Scale bar = 25 µm. (TIF) [file pone.0054809.s001.tif]

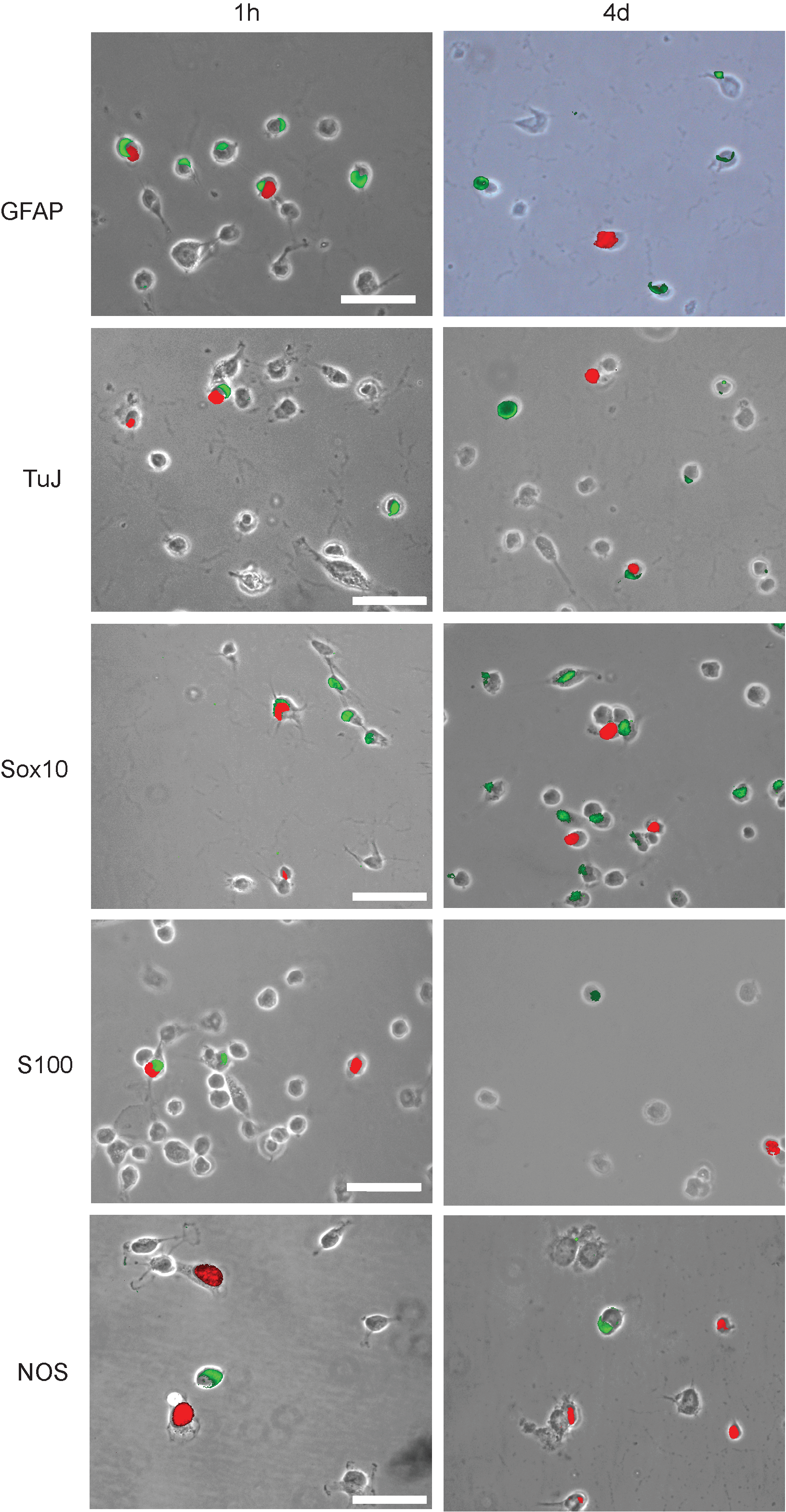

Supplement: Figure S2 — Double labeling of neurosphere cells for neural cell markers and EdU incorporation. Primary mouse neurospheres previously cultured for 15 days under non-adherent conditions were labeled with a 1 h pulse of 10 µM EdU. The neurospheres were then dissociated and allowed to attach after which they were fixed and permeabilized before immunostaining for the neural cell markers shown and processing to reveal EdU incorporation. The montages shown were constructed in Adobe Photoshop from 3 separate images captured to demonstrate the EdU incorporation, immunofluorescence and phase contrast images. Scale bars = 25 µm. (TIF) [file pone.0054809.s002.tif]

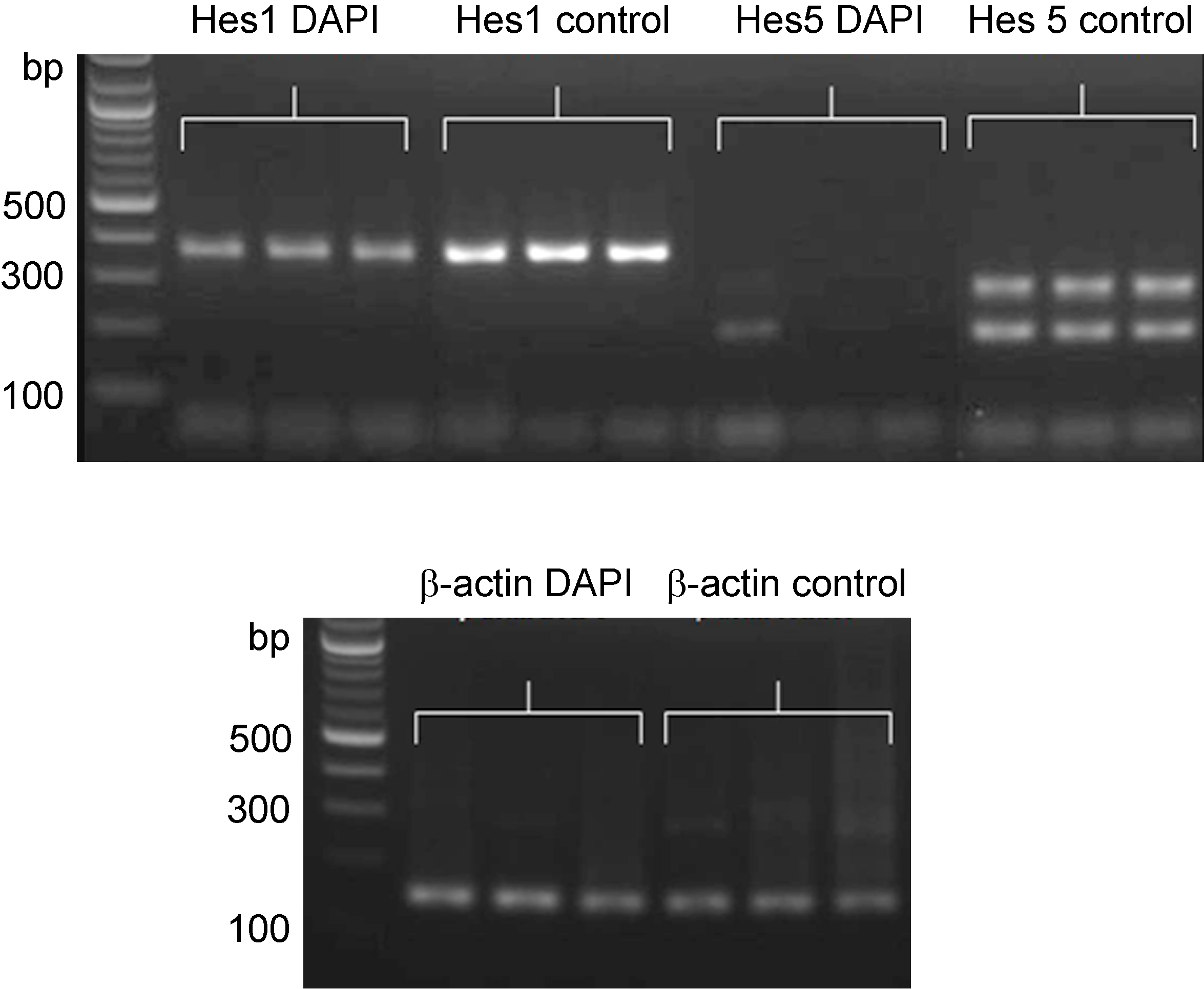

Supplement: Figure S3 — Agarose gel electrophoresis of qPCR products after DAPT treatment of neurospheres. The PCR products obtained from the experiment in Fig. 5A was electrophoresed in 2% agarose gels. Calibration standards (bp) are shown on the left hand side of each gel. PCR product sizes were: Hes1 = 354 bp, Hes5 = 183 bp and 269 bp and β-actin = 143 bp. The DNA in each excised band was sequenced to confirm PCR product identity; the double bands for Hes5 represent two splice variants amplified by the primer pair used. (TIF) [file pone.0054809.s003.tif]

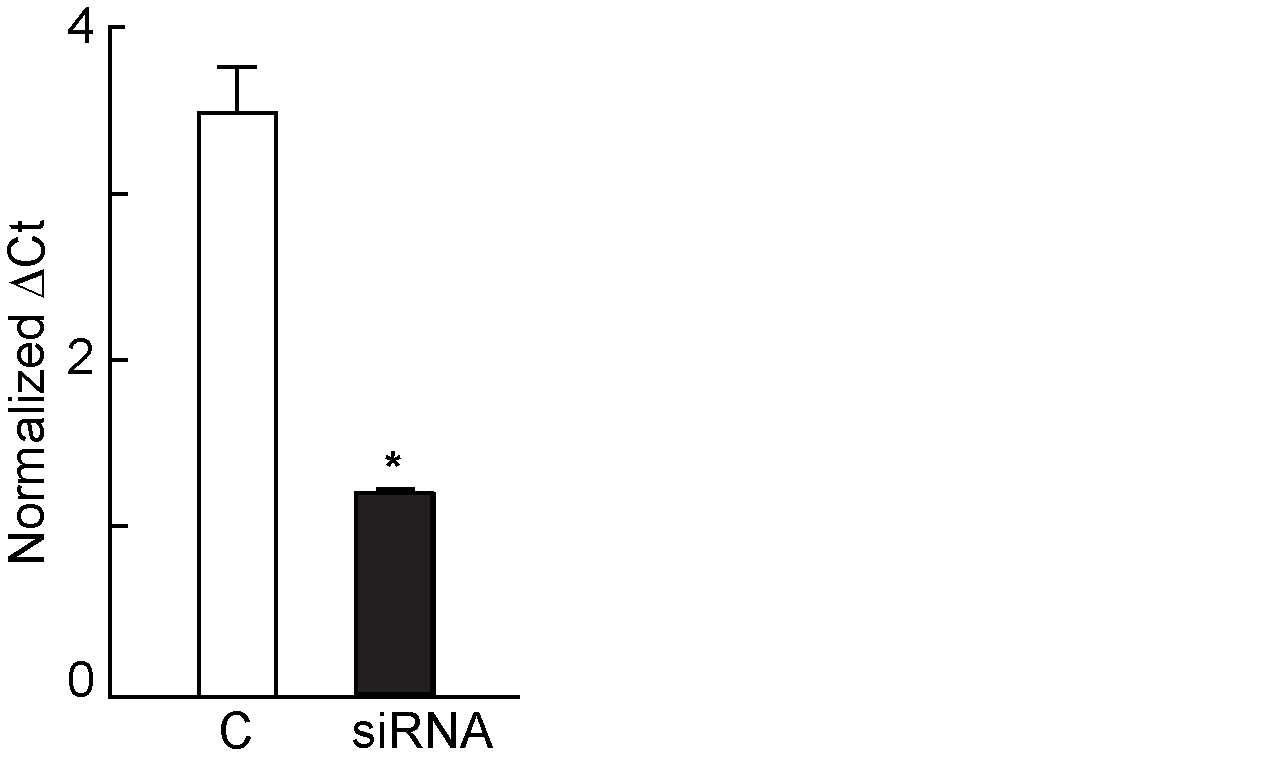

Supplement: Figure S4 — Confirmation of RBPjκ knockdown in human neurospheres. Mature 2nd to 3rd passage human neurospheres were dissociated and cultured on fibronectin coated chamber slides for 96 h. The dissociated cells were transfected with HsRBPJ_3 siRNA knockdown specific for RBPjκ or a corresponding negative control. Levels of RBPjκ were determined by qPCR. Columns show the normalised ΔCt values (± SEM, n = 3). * = P<0.01. (TIF) [file pone.0054809.s004.tif]
